# Toll-Like Receptor 11 (TLR11) Interacts with Flagellin and Profilin through Disparate Mechanisms

**DOI:** 10.1371/journal.pone.0148987

**Published:** 2016-02-09

**Authors:** Hirotsugu Hatai, Alice Lepelley, Wangyong Zeng, Matthew S. Hayden, Sankar Ghosh

**Affiliations:** 1 Department of Microbiology & Immunology, College of Physicians & Surgeons, Columbia University, New York, New York, United States of America; 2 Department of Pathology, Immunology and Microbiology, Graduate School of Medicine, The University of Tokyo, Bunkyo-ku, Tokyo, Japan; 3 Department of Dermatology, College of Physicians & Surgeons, Columbia University, New York, New York, United States of America; University at Buffalo, UNITED STATES

## Abstract

Toll-like receptors (TLRs) are innate immune receptors that sense a variety of pathogen-associated molecular patterns (PAMPs) by interacting with them and subsequently initiating signal transduction cascades that elicit immune responses. TLR11 has been shown to interact with two known protein PAMPs: *Salmonella* and *E*. *coli* flagellin FliC and *Toxoplasma gondii* profilin-like protein. Given the highly divergent biology of these pathogens recognized by TLR11, it is unclear whether common mechanisms are used to recognize these distinct protein PAMPs. Here we show that TLR11 interacts with these two PAMPs using different receptor domains. Furthermore, TLR11 binding to flagellin and profilin exhibits differential dependency on pH and receptor ectodomain cleavage.

## Introduction

TLRs recognize microbial molecules termed PAMPs by interacting with them in the extracellular compartment or within endosomes [1]. Following interaction with their ligands, TLRs initiate signal transduction cascades leading to activation of immune cells and antimicrobial defense [1].

TLRs are type I transmembrane proteins that possess an N-terminal ectodomain (ECD) containing leucine-rich-repeat (LRR) motifs for ligand interaction, a single transmembrane domain, and a C-terminal cytoplasmic signaling domain [1]. There are 13 TLRs in mammalian species and each TLR recognizes specific ligands [1]. TLR11 is known to recognize *Toxoplasma gondii* (*T*. *gondii*) profilin (TPRF) [2]. Recently, we reported that TLR11 also recognizes flagellin (FliC) from *E*. *coli* and *Salmonella typhimurium* [3].

Flagellin is a bacterial protein that forms flagella, the structure that promotes bacterial chemotaxis and invasion in host tissues [4]. Recognition of flagellin by the mammalian host is an important event in mounting immune responses to flagellated bacteria. However, the consequence of flagellin recognition on infection is complex. For example, although TLR5, a known flagellin receptor, recognizes and responds to *Salmonella* flagellin, and induces proinflammatory cytokines, TLR5-deficient mice show enhanced resistance against oral *Salmonella* infection [5]. In addition to infectious diseases, the immune response to flagellin has also been implicated in autoimmune diseases. Flagellin is an immunodominant antigen in murine colitis and human Crohn’s disease [6]. Finally, as the best characterized protein PAMP, flagellin is also being actively investigated for use as a vaccine adjuvant [7].

*T*. *gondii* is a protozoan apicomplexan parasite that can infect all mammals. In humans, infection primarily occurs through the ingestion of infected food [8]. In healthy adults, the immune system controls *T*. *gondii* and maintains it in a quiescent state [8]. However, *T*. *gondii* causes severe neurological disease in immunocompromised individuals as well as when transmitted in utero [8].

TLR11 recognizes the unconventional apicomplexan actin-binding protein profilin [8], which regulates parasite motility and host cell invasion [9]. Studies of TLR11 function in *T*. *gondii* infection have reinforced the importance of parasite recognition and T helper 1 cells (Th1) response in *T*. *gondii* clearance in murine models [8].

Although TLR11 is not expressed in humans [10], investigation of TLR11 informs our understanding of the human immune response against bacterial and apicomplexan pathogens carrying these two PAMPs because TLR11 regulates immune responses which are shared with other TLRs [2,3,10,11]. In addition, TLR11-deficient mice are susceptible to human pathogens carrying these two PAMPs and may therefore serve as animal models to examine corresponding human infectious diseases [2,3,10,11]. Furthermore, the established ability of TLR11 to recognize two distinct protein PAMPs provides a unique opportunity to further our understanding of recognition of protein PAMPs by pattern recognition receptors. However, it is first necessary to understand the mechanism of ligand recognition by TLR11. Indeed, the mechanism by which TLR11 separately senses these two distinct PAMPs is completely unknown. Therefore, in this study, we have examined both the biochemical requirements and protein domains required for TLR11 interaction with these two distinct PAMPs.

Here we show that TLR11 ectodomain (ECD) is cleaved in a cathepsin-dependent manner, similar to other intracellular TLRs. The C-terminal region of cleaved-TLR11 interacts strongly with FliC but not with TPRF. In contrast to TLR11 binding to TPRF, acidic pH conditions significantly promoted the interaction between TLR11 and FliC. Furthermore, TLR11 used different protein domains to interact with TPRF and FliC. These results demonstrate that a single TLR is capable of binding to distinct PAMPs through highly divergent mechanisms and suggest that the modular structure of the TLR ectodomain is compatible with recognition of unique PAMPs.

## Materials and Methods

### Plasmid constructs

The plasmids coding for mouse TLR11-FLAG was previously described [12]. Mouse TLR5 cDNA was amplified from the TLR5 vector [3] by PCR and cloned into p3xFLAG-CMV-14 (Sigma) (TLR5-FLAG). Primer sequences are as follows: forward (5’- atagcggccgcccataggatcatggcatgtcaac -3’) and reverse (5’- accgatatcgcggaaatggttgctatggttc -3’). Human TLR2 cDNA was amplified from U937 cell cDNA and cloned into p3xFLAG-CMV-14 (TLR2-FLAG). Primer sequences are as follows: forward (5’- atagcggccgccactggacaatgccacatactttg—3’) and reverse (5’- cgcggatccggactttatcgcagctctcag -3’). The mutated and truncated TLR11-FLAG constructs generated from TLR11-FLAG by using circular mutagenesis PCR. Primer sequences are as follows: Y39A, forward (5’- cctggtctcccgcgctttcacattctgc -3’) and reverse (5’- gcagaatgtgaaagcgcgggagaccagg -3’); C43A, forward (5’- gctatttcacattcgcccgccactccaagc -3’) and reverse (5’- gcttggagtggcgggcgaatgtgaaatagc -3’); R44A, forward (5’- ctatttcacattctgcgcccactccaagctatc -3’) and reverse (5’- gatagcttggagtgggcgcagaatgtgaaatag -3’); Δ668–709, forward (5’- gagccatgggttaagctctttttggctagttctgccttggtgttcatgc -3’) and reverse (5’- actagccaaaaagagcttaacccatggctccatccacgcattggcacag -3’); Δ643–709, forward (5’- tacagccctcagatgctctttttggctagttctgccttggtgttcatg -3’) and reverse (5’- actagccaaaaagagcatctgagggctgtactccctcagactgtcctg -3’). To construct the vectors encoding the chimeras between TLR11 and TLR2, ClaI site was inserted into TLR11-FLAG and TLR2-FLAG by circular mutagenesis PCR and subsequently the corresponding cDNA regions were amplified by PCR and ligated. Primers sequences are as follows: ClaI site insertion in TLR11-FLAG, forward (5’- caggaggctctggcttcctgccacatcgatagcctgaagaccttgggtctttcaag -3’) and reverse (5’- cttgaaagacccaaggtcttcaggctatcgatgtggcaggaagccagagcctcctg -3’); TLR2 LRR10—C-terminus, forward (5’- cacatcgatggtaattttagagcatctgataatg -3’) and reverse (5’- cgcggatccggactttatcgcagctctcag -3’); ClaI site insertion in TLR2-FLAG, forward (5’- gaggatgcctggccctctctacaaatcgatactttaattttaaggcaaaatcatttg -3’) and reverse (5’- caaatgattttgccttaaaattaaagtatcgatttgtagagagggccaggcatcctc -3’); TLR11 LRR14—C-terminus, forward (5’- caaatcgatcaactagagaccttgaagctg -3’) and reverse (5’- ccgggatccccctagcctgctcctcagcc -3’). To construct FliC protein expression vector, corresponding DNA sequence was amplified by PCR from *Typhimurium* SL1344 (ATCC) genomic DNA and cloned into pET15 vector (Novagen). Primers sequences are as follows: forward (5’- aaactcgagatggcacaagtcattaataca -3’) and reverse (5’- aaaggatccttaacgcagtaaagagaggac -3’).

### Recombinant Histidine-tagged TPRF protein expression and purification

Histidine-tagged *T*. *gondii* profilin protein (His-TPRF) was recombinantly expressed in ScarabXpress T7 lac Chemically Competent Cells (Scarab Genomics), lacking flagellar operons [13], by transformation using the pET28 vector [12]. The purification steps were previously described [12].

### Recombinant Histidine-tagged FliC protein expression and purification

Histidine-tagged FliC protein (His-FliC) was recombinantly expressed in ScarabXpress T7 lac Chemically Competent Cells. Cultures were shaken at 37°C to an OD600 of 0.6. His-FliC expression was then induced using 0.4 mM IPTG at 27°C overnight. The culture was pelleted and lysed in PBS (pH 7.4) (GIBCO) containing 5% glycerol, 0.5% Triton-X and 1 mM PMSF on ice. The remaining purification steps were preformed as for His-TPRF, except for the purification step using Superdex 200 10/30 size-exclusion column.

### His-TPRF pulldown assay

HEK-293T cells or Hela cells were plated in a 6-well plate and transiently transfected with vectors encoding the indicated TLRs using either Lipofectamine 2000 (Invitrogen) or polyethylenimine (Polysciences). After 2 days, cells were detached, washed twice with PBS and pelleted by centrifugation. Cell pellets were lysed with lysis buffer containing 1% Nonidet P-40, 150 mM NaCl, protease inhibitors (1 mM PMSF, 1.5 μg/ml aprotinin, 1 μg/ml leupeptin, and 1 μg/ml pepstatin) and either 30 mM 2-Morpholinoethanesulfonic acid (MES) (pH 6.0) or 25 mM HEPES (pH 7.0) on ice. Cell lysates were clarified by ultra-centrifugation at 45,000 x g for 20 min at 4°C. The concentration of Nonidet P-40 in the cleared cell lysates was diluted to 0.5% by addition of buffer containing 150 mM NaCl, protease inhibitors and either 30 mM MES (pH 6.0) or 25 mM HEPES (pH 7.0). Cell lysates were mixed with 15 uL TALON Metal Affinity Resin (Clontech) and incubated at 4°C to remove non-specific binding proteins. The resulting pre-cleared lysates were collected by centrifugation at 4°C and mixed with 3 μg/ml His-TPRF. After 4–6 h incubation with rotation at 4°C, mixtures were rotated with 2 mM imidazole and 15 uL TALON Metal Affinity Resin for 20 min at 4°C. Resins were collected by centrifugation, resuspended and incubated for 2 min with washing buffer containing 0.5% Nonidet P-40, 150 mM NaCl, 20 mM imidazole and either 30 mM MES (pH 6.0) or 25 mM HEPES (pH 7.0) on ice. This washing step was repeated three times. Resins were boiled for 5 min in SDS sample buffer containing 50 mM Tris-HCl (pH 6.8), 2% sodium dodecyl sulfate, 10% glycerol and 2% β-mercaptoethanol.

### His-FliC pulldown assay

Cell lysates were prepared as for TPRF pulldown assay. Cell lysates were mixed with 20 ul Glutathione Sepharose 4B (GE healthcare) beads and incubated at 4°C to remove non-specific binding proteins. The resulting pre-cleared cell lysates were collected by centrifugation at 4°C and mixed with 3 μg/ml His-FliC. After 90 min rotation at 4°C, mixtures were rotated with 20 ul anti-FliC mouse monoclonal antibody (Invivogen) -conjugated Glutathione Sepharose 4B beads for 3 h at 4°C. Beads were collected by centrifugation and washed with washing buffer containing 0.5% Nonidet P-40, 150 mM NaCl and either 30 mM MES (pH 6.0) or 25 mM HEPES (pH 7.0). This washing step was repeated three times. Beads were boiled for 5 min in SDS sample buffer.

### Western blots

Samples were separated using 10% polyacrylamide gels. After electrophoresis, proteins were transferred to polyvinylidene fluoride membrane (Millipore). The membrane was blocked with 5% skim milk in TTBS and incubated with the following primary antibodies: 1/1000 anti-FLAG M2 (SIGMA-ALDRICH, F1804), 1/5000 anti-polyHistidine (SIGMA-ALDRICH, H1029) or 1/500 anti-FliC (Invivogen, mabg-flic) mouse monoclonal antibodies. An appropriate HRP-conjugated secondary antibody was added and HRP activity was detected using Luminata Forte Western HRP substrate (Millipore). Densitometric analysis was performed using ImageJ [14]. Statistical significance was determined using the Students *t* test.

## Results

### TLR11 ECD cleavage is sensitive to cathepsin inhibition

**Fig 1:**
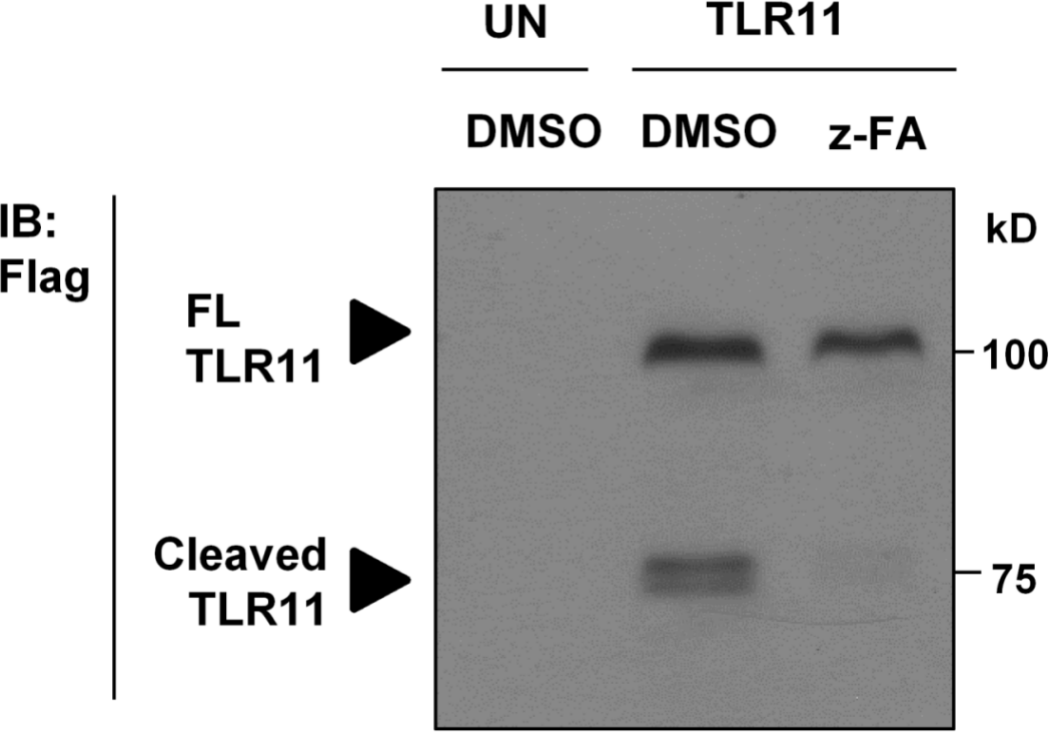
TLR11-Flag is cleaved and its cleavage is sensitive to cathepsin inhibition. HEK-293T cells were transiently transfected with C-terminal Flag-tagged TLR11. Cells were treated with either DMSO or 20 uM z-FA-fmk (z-FA) before lysis. Lysates were analyzed by Western blot with anti-Flag antibody: full-length (FL), immunoblotting (IB), untransfected cells (UN).

In order to investigate in vitro ligand binding by TLR11, we used HEK-293T cells to express epitope tagged full length TLR11. We then analyzed TLR11 expression by Western blotting. Consistent with previous reports [15], upon overexpression of TLR11-Flag in HEK-293T cells, TLR11-Flag was present in both full-length (FL) and its cleaved forms, as seen by Western blotting (Fig 1).

Cleavage of the ectodomain of other intracellular TLRs depends on cathepsin activity in endosomes [16,17]. Therefore, we examined the sensitivity of TLR11-Flag cleavage to cathepsin inhibition. We overexpressed TLR11-Flag in HEK-293T cells by transfection and inhibited cathepsin activity using Z-Phe-Ala fluoromethyl ketone (z-FA-fmk), which was shown to impair the cleavage of both TLR3 and TLR9 [16,17]. Similar to these intracellular TLRs, z-FA-fmk treatment abrogated the cleavage of TLR11-Flag (Fig 1). Thus TLR11 ECD cleavage is cathepsin-dependent.

### Cleaved TLR11-Flag strongly interacts with His-FliC but not His-TPRF

The presence of both cleaved and uncleaved forms of TLR11 in lysates from transfected HEK-293T cells (Fig 1) allowed us to investigate which form of TLR11 is responsible for ligand interaction. To do so, we overexpressed TLR11-Flag in HEK-293T cells, lysed the cells, added either His-FliC or His-TPRF to the lysates, and used either anti-flagellin monoclonal antibody conjugated G-sepharose beads to pull down His-FliC or TALON resin to pull down His-TPRF.

**Fig 2:**
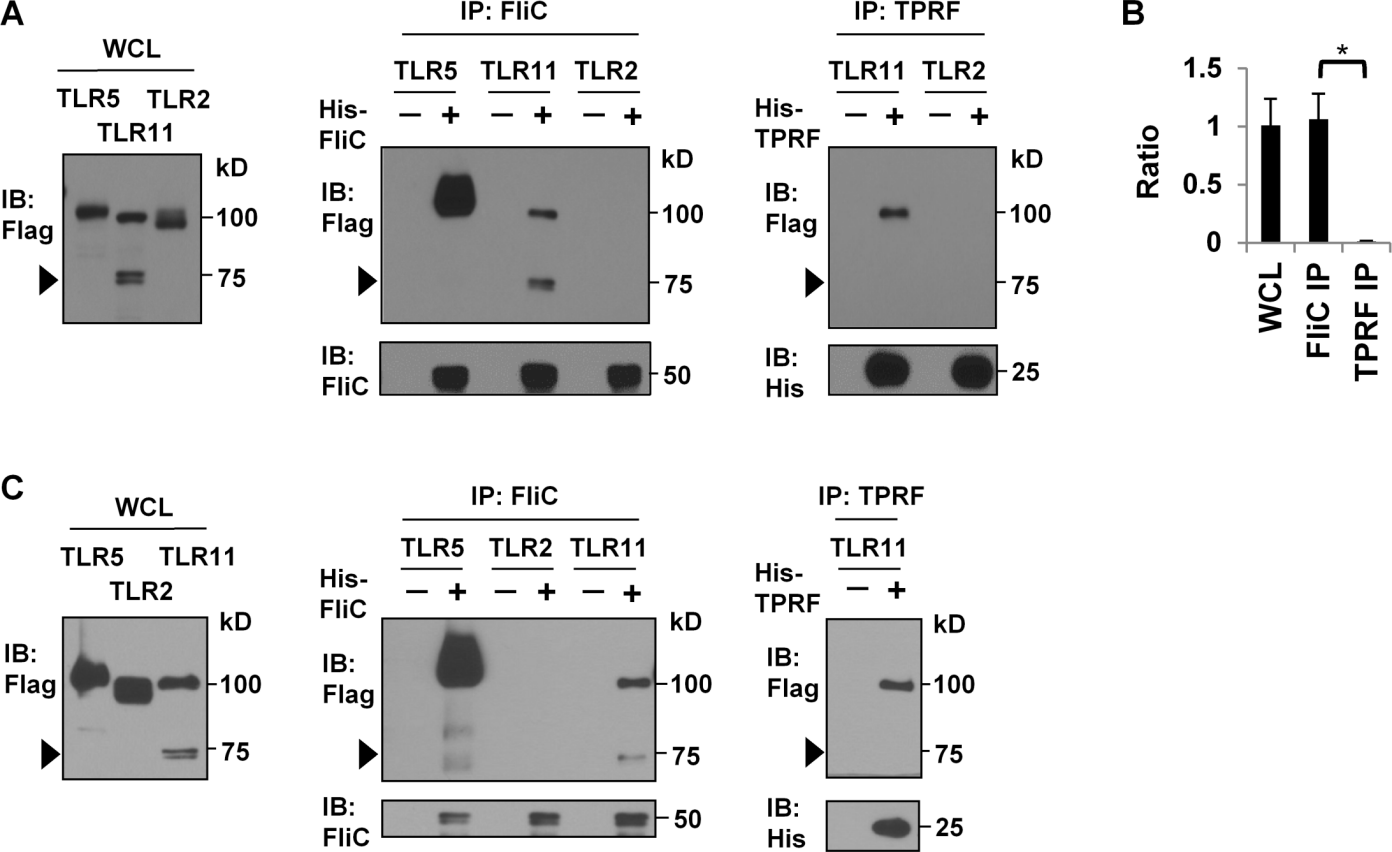
Cleaved TLR11-Flag strongly interacts with His-FliC but not His-TPRF. (A) Pulldown assay at pH6.0. HEK-293T cells were transiently transfected with C-terminal Flag-tagged TLR and cleared lysates were incubated with the indicated ligand at pH 6.0. His-TPRF and His-FliC were pulled down by TALON resin and anti-FliC antibody conjugated G-sepharose beads, respectively. TLR binding was analyzed by Western blots. Arrow heads indicate the position of cleaved TLR11-Flag band. Data are representative of at least 10 independent experiments; immunoprecipitation (IP). (B) Ratio of the C-terminal fragment of cleaved TLR11 to full-length TLR11 was determined by densitometric analysis of Western blots using ImageJ. Data represents the mean, with error bars indicated standard deviation, from seven independent experiments by using HEK-293T cells. *p < 0.0001, FliC IP versus TPRF IP.

Notably, His-FliC strongly interacted with both full-length and the C-terminal fragment of cleaved TLR11-Flag (Fig 2A). In contrast, His-TPRF strongly interacted with full-length TLR11-Flag, but the interaction with the C-terminal fragment of cleaved TLR11-Flag (Fig 2A and 2B), or the negative control, TLR2, was not detectable (Fig 2A). Thus only full-length TLR11-Flag interacts with His-TPRF, while both cleaved and full-length TLR11-Flag binds to His-FliC.

### Acidic conditions are required for TLR11-FliC interaction

Intracellular TLRs, TLR3 and TLR9, interact with their ligands at acidic pH, found within the endolysosomal system [18,19]. His-FliC interacted with both full-length and cleaved TLR11-Flag, similarly to other intracellular TLR ligand interactions, we therefore examined the effect of acidic pH on TLR11-FliC interaction.

**Fig 3:**
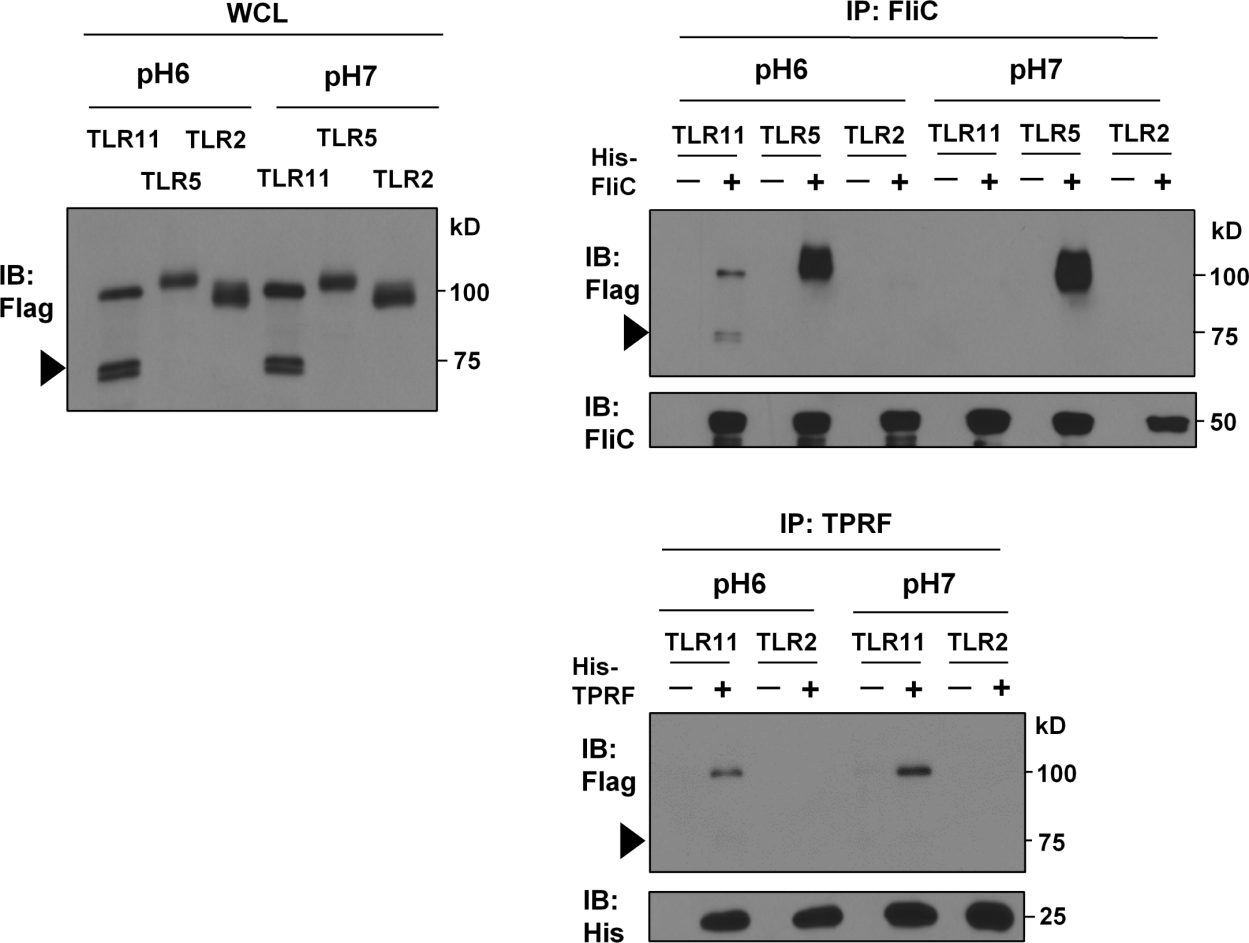
Acidic conditions are required for TLR11-FliC interaction. Pulldown assay at either pH 6.0 or pH 7.0, These experiments were performed like in Fig 2A with changing pH condition. Data are representative of six independent experiments.

As expected, the interaction of TLR11-Flag with His-FliC was detected only at acidic conditions (pH 6.0) (Fig 3) but not at neutral conditions (pH 7.0) (Fig 3). In contrast, both the interaction of TLR11-Flag with His-TPRF and the interaction of TLR5-Flag with His-FliC were strongly detected at both acidic and neutral conditions (Fig 3). Therefore, TLR11 exhibits distinct biochemical requirements for binding to two different protein PAMPs, and two different TLRs, TLR11 and TLR5, have distinct requirements for binding to the same PAMP.

### Both N- and C-terminal regions of TLR11 can interact with FliC individually

**Fig 4:**
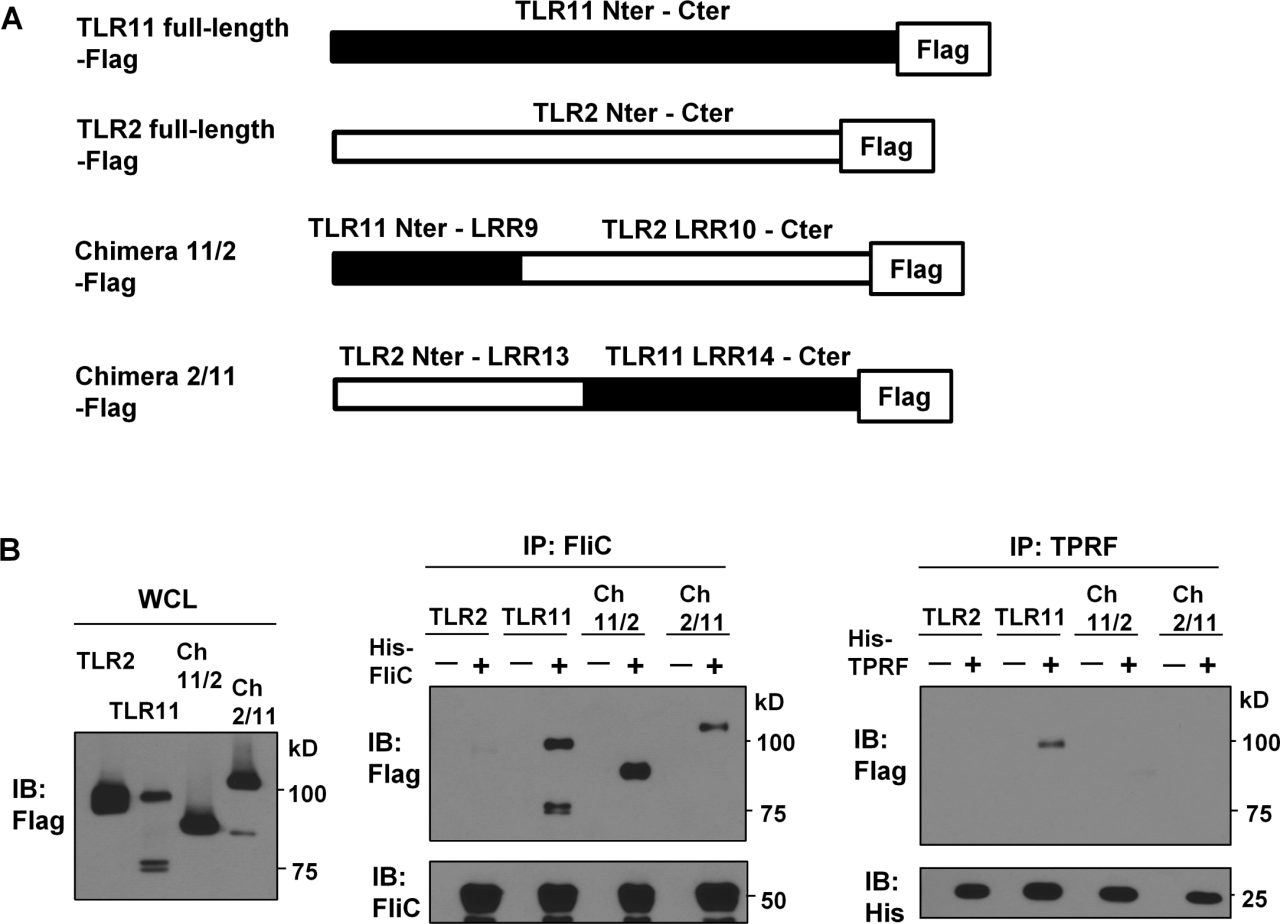
Both N- and C-terminal regions of TLR11 can interact with FliC individually. (A) Schematic representation of WT and chimeric TLR-Flag constructs. The black regions are from TLR11 and the gray regions are from TLR2. (B) His-TPRF pulldown assay at pH 6.0. Hela cells were transiently transfected with C-terminal Flag-tagged TLR2, TLR11 or TLR2/11 chimera. These experiments were performed as in Fig 2A: N-terminus (Nter), C-terminus (Cter), TLR11/2 chimera (Ch11/2), TLR2/11 chimera (Ch2/11). Data are representative of three independent experiments.

Considering the differences between TLR11-Flag binding to His-TPRF and His-FliC, we hypothesized that TLR11 uses different protein domains to interact with TPRF and FliC. To better understand these differences, we constructed a series of chimera between TLR11 and TLR2 (Fig 4A). Using these chimeric receptors, we investigated which regions of TLR11 are required for interaction with His-TPRF and His-FliC, respectively.

We used the TLR11/2 chimera consisting of TLR11 N-terminus—LRR9 and TLR2 LRR10—C-terminus and the TLR2/11 chimera consisting of TLR2 N-terminus—LRR13 and TLR11 LRR14—C-terminus. Surprisingly, both of the chimeras interacted with His-FliC but neither was able to pull down His-TPRF (Fig 4B). Therefore, TLR11 uses different domains to interact with either TPRF or FliC.

### Mapping both the N- and C-terminal regions of TLR11 motifs required for His-TPRF interaction

Given that both the TLR11/2 and TLR2/11 chimeras bind to FliC, it appears that they are capable of binding to ligand. Therefore, consistent with the failure of cleaved TLR11 to bind TPRF, it seems that both the N- and C-terminal region of TLR11 are critical for interaction with TPRF. To further delineate the contribution of the N- and C-terminal LRRs, we performed targeted mutagenesis of candidate motifs within the TLR11 ECD.

**Fig 5:**
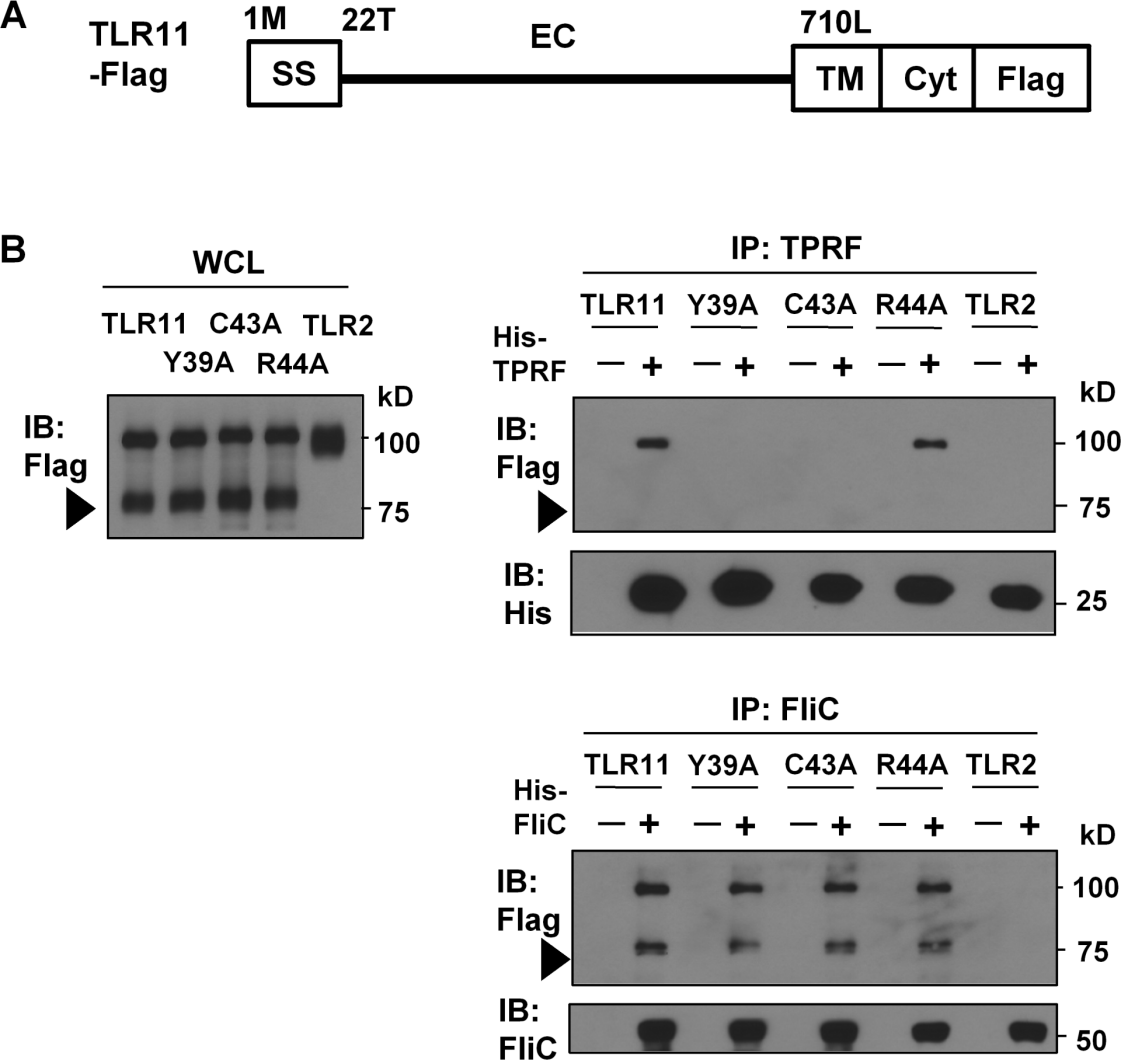
Mutations in the N-terminal region of TLR11 impair His-TPRF binding. (A) Schematic representation of TLR11 domains: signal sequence (SS), ectodomain (EC), transmembrane domain (TM), cytoplasmic domain (Cyt). Numbers represent the putative initial amino acid of each domain. (B) His-TPRF pulldown assay at pH6.0. HEK-293T cells were transiently transfected with C-terminal Flag-tagged TLR11 or its mutants. These experiments were performed as in Fig 2A. Arrow heads indicate the position of cleaved TLR11-Flag band. Data are representative of four independent experiments.

Based on a comparative sequence analysis of TLR11 (11), amino acids M1 to S21 correspond to the signal sequence and amino acids T22 to S709 comprise the extracellular domain of the receptor consisting of 26 LRRs (LRR-NT, LRR 1–24, LRR-CT) (Fig 5A).

To examine the role of the N-terminal region of the TLR11 ECD on TPRF interaction, we therefore constructed TLR11-Flag mutants in which we deleted portions of the N-terminal LRRs of the TLR11 ECD. Based on the loss of TPRF binding observed upon deletion of N-terminal LRRs (data not shown), we constructed a series of alanine point mutations in LRR-NT of TLR11-Flag to define which residues are critical for TPRF interaction. As shown in Fig 5B, mutation of Y39 or C43 to alanine severely impaired His-TPRF interaction, without affecting TLR11 cleavage (Fig 5B). In contrast, mutation of R44 to alanine did not dramatically change His-TPRF interaction. Interestingly, neither the Y39A or C43A mutant impaired His-FliC interaction (Fig 5B). Therefore, Y39 and C43 in LRR-NT are critical for His-TPRF interaction but not His-FliC interaction. Thus the N-terminal region of the TLR11 ECD plays a critical role in TPRF binding.

**Fig 6:**
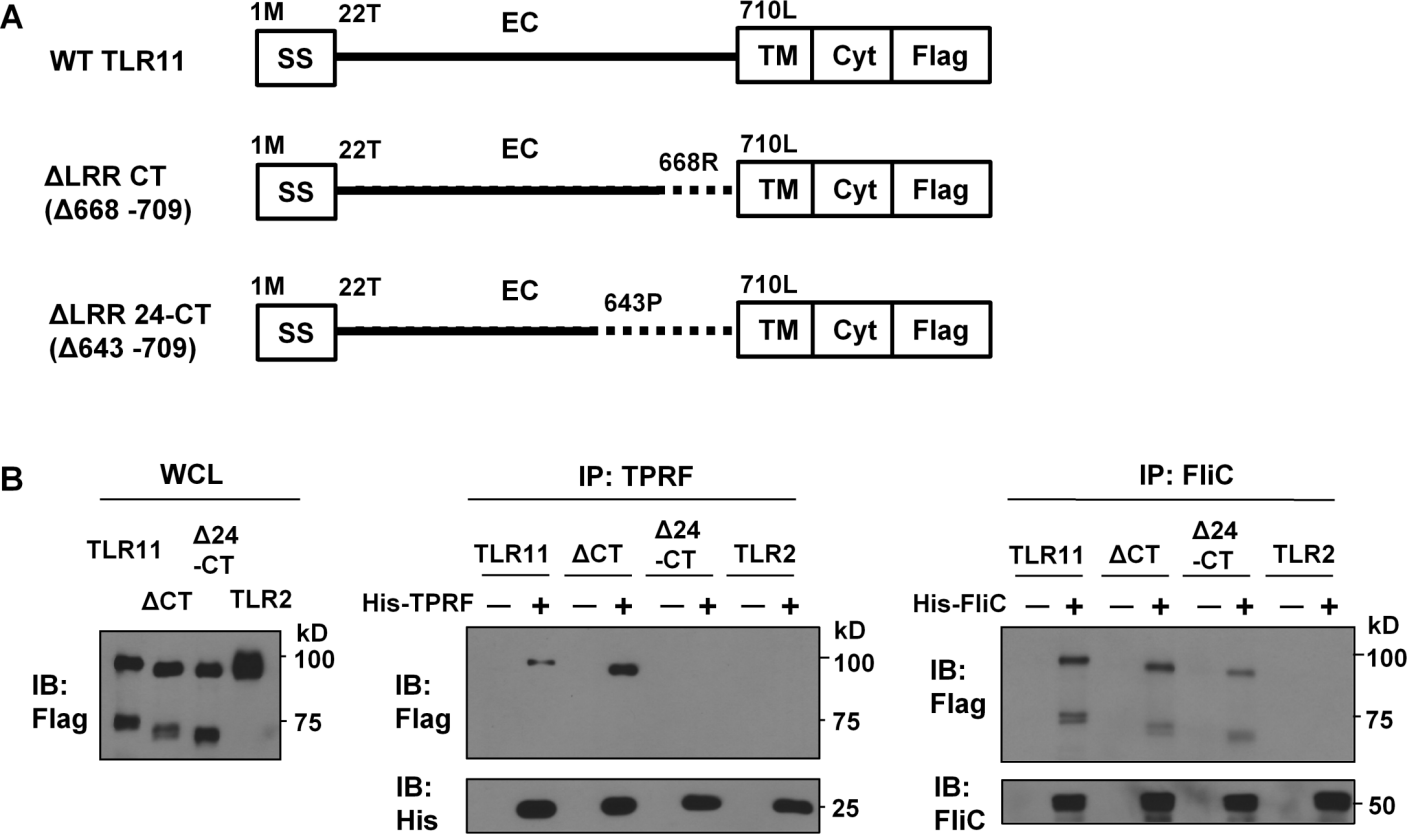
Deletion of C-terminal LRRs of TLR11-Flag impair His-TPRF interaction. (A) Schematic representation of WT or truncated-TLR11-Flag. Numbers represent the initial amino acid of deleted region or a domain. (B) His-TPRF pulldown assay at pH 6.0. HEK-293T cells were transiently transfected with C-terminal Flag-tagged TLR11 or truncated mutants. These experiments were performed like in Fig 2A. Data are representative of three times experiments.

To examine the role of the C-terminal region of the TLR11 ECD on TPRF interaction, we also constructed a series of truncated TLR11-Flag mutants in which we deleted portions of the C-terminal region of the TLR11 ECD (Fig 6A). Removal of the 66 amino acids in the C-terminal ECD (Δ643–709, corresponding to LRR24-CT) severely impaired His-TPRF interaction (Fig 6B), but a truncation of 41 amino acids (Δ668–709, corresponding to ΔLRR-CT) did not. However, both Δ643–709 and Δ668–709 strongly interacted with His-FliC (Fig 6B). Therefore LRR24 is critical for His-TPRF interaction but not His-FliC interaction.

## Discussion

This study shows that TLR11 specifically interacts with two protein PAMPs, TPRF and FliC, using distinct mechanisms that depend on receptor cleavage, pH and different receptor domains.

It has previously been reported that TLR cleavage affects ligand interaction. Cleaved C-terminal fragment of TLR9 binds its ligand more strongly than full-length TLR9 [16,20]. Surprisingly, TLR11 completely lost its ability to bind to TPRF upon ECD cleavage (Fig 2). Therefore, the ability of TLR11 to recognize different PAMPs can be altered by post-translational receptor modification.

It has also been reported that ligand recognition by intracellular TLRs is pH-dependent [18,19]. One possible explanation for this dependence is that acidic pH conditions provide essential electric charge for the interaction, as shown for the interaction of TLR3 with dsRNA [19]. It seems that FliC recognition by TLR11 is more restricted than FliC recognition by TLR5: TLR5 strongly interacted with FliC at both acidic and neutral pH conditions whereas acidic conditions are required for TLR11-FliC interaction (Fig 3).

TLR11 interacted specifically with these two distinct PAMPs through different domains. Recently, the precise role of N-terminal fragments of intracellular TLRs has been a controversial topic. It was previously thought that N-terminal fragment of TLR9 does not play an important role in ligand recognition [16]. In contrast, two articles have recently reported a critical role for N-terminal fragment of TLR9 [21,22]. Other than TLR9, the N-terminal fragment of other intracellular TLRs such as TLR3, TLR7 and TLR8 also plays a critical role in ligand recognition [23–26]. Similarly, the TLR11 mutagenesis reported here supports a critical role for the N-terminal region of intracellular TLRs in ligand recognition by the non-cleaved, full-length form of the receptors.

**Fig 7:**
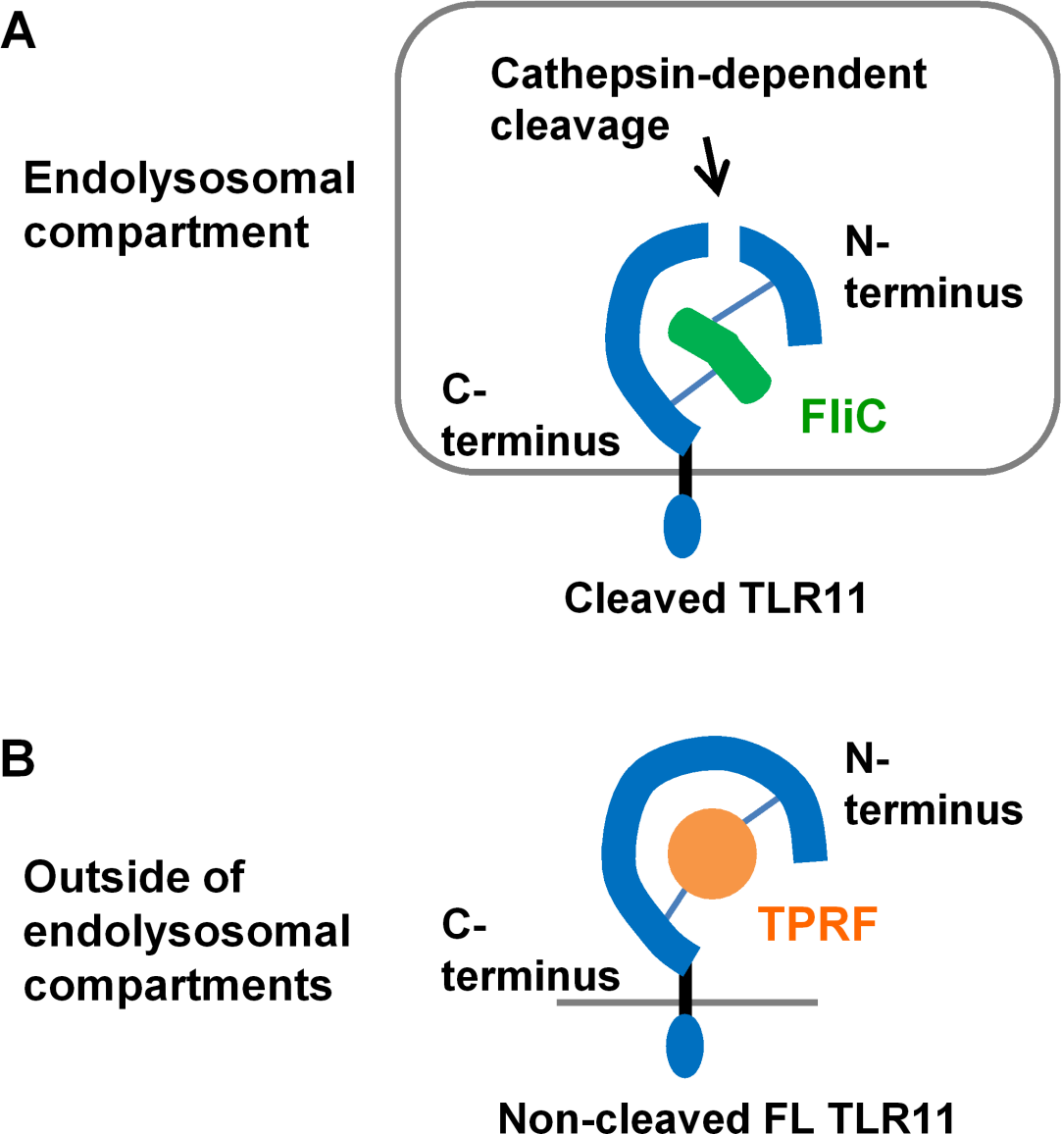
Putative model of TLR11 ligand recognition. (A) Cathepsin-derived C-terminal region of TLR11 interacts with FliC within an acidic endolysosomal compartment. Both N- and C-terminal LRRs of TLR11 can interact with FliC independently. (B) Non-cleaved, full-length TLR11 interacts with TPRF outside the lysosomal compartment. Both the N- and C-terminal LRRs of TLR11 are required for TPRF interaction.

Considering both the acidic pH-dependency (Fig 3) and interaction of the cathepsin-derived C-terminal region with His-FliC (Fig 2), TLR11 is predicted to preferentially recognize FliC in the endolysosomal compartment (Fig 7A). In contrast, interaction of full-length TLR11-Flag with His-TPRF was strongly detected at neutral conditions (Fig 3). Consistent, with these findings, binding of TPRF was also shopwn with the uncleaved extracellular domain of TLR11, although it was reported that this binding occurred more weakly at pH8 than at pH 6.0 [27]. Since the cathepsin-derived C-terminal region of TLR11-Flag does not interact with His-TPRF, TLR11 should recognize TPRF prior to cleavage within the endoplysosomal system (Fig 7B).

Hence TLR11 might utilize distinct subcellular compartments to sense different PAMPs. This putative TLR11 ligands recognition model is consistent with some previous reports. TLR11 is highly expressed in epithelial cells in various organs such as intestine, lung and skin [28] and prevents *Salmonella* invasion [11]. Therefore compartmentalization of TLR11 recognition of flagellin might lead to both tolerance to commensal bacterium-derived flagellin and efficient response to invasive *Salmonella*. In contrast to FliC, it seems that TPRF recognition happens outside of the lysosomal compartments. Actually, it is thought that TLR11 recognizes TPRF before parasite invasion because direct contact with *T*. *gondii* is not required for TLR11-dependent IL-12 production [29]. Furthermore, the intact acidic loop and β-hairpin of TPRF are required for TLR11-dependent IL-12 production [30] and these structures are likely unstable within the lysosome.

It was thought that UNC93B1 only regulated endosomal TLRs. Thus given that recognition of TPRF by TLR11/12 required UNC93B1 [29], it was previously suspected that this recognition occurs within an endosomal compartment. However, it was recently shown that UNC93B1 also regulates the cell surface expression of both TLR5 and TLR9 [22,31]. Hence, the requirement for UNC93B1 for the cellular response to *T*. *gondii* profilin does not necessarily mean that TLR11 recognizes TPRF only in endolysosomal compartments or only after cleavage. These data leave open the possibility of TPRF binding to full length TLR11 in a neutral pH environment, either at the cell surface or within early endosomses. Alternatively, it is possible that in the context of a TLR11/12 heterodimer, that LRR24 TPRF binding motif is sufficient for TPRF recognition, or that the pH dependence of the TPRF response might differ from that observed using in vitro assays. Consequently, in the future, it will also be important to determine whether both N- and C-terminal extracellular domains of TLR11 are required for TPRF recognition by the murinme innate immune system in the context of a TLR11/12 heterodimer.

Unfortunately, such functional studies are not readily performed through simple transfection experiments as the requirements for signaling by TLR11, in response to either PAMP, remain poorly understood. Experiments using transfection of TLR11 together with a reporter gene into various cell lines yield weak responses to either PAMP. For example, in CHO cells or HEK-293 cells expressing TLR11, TPRF stimulation resulted in modest activation of an NF-κB reporter [2,12]. Similar studies of TLR11 recognition of FliC also produced only weak activation of an NF-κB reporter gene [3,10]. Subsequently it was found that innate recognition of TPRF through TLR11 requires the presence of TLR12 [12]. However, even with co-expression of both TLR11 and TLR12, the response of NF-κB reporter genes to TPRF is relatively poor [12,27]. Whether or not there are additional co-factors required for recognition of FliC by TLR11 remains to be determined. Consequently, it will be necessary to investigate the role of specific TLR11 domains in the TPRF and FliC response through genetic engineering of murine innate immune cells. Such future studies will be needed in order to discriminate between the requirements for binding, described here, and the domains of TLR11 that are required for signaling with, or independent of, TLR12. Future work should use mouse models to address how the requirements for binding to TPRF and FliC impact signaling through TLR11 and whether the requirements for TLR11-dependent signaling in response to TPRF and FliC, independent of bPAMP binding, are also distinct.
